# The Association between Negative and Dysexecutive Syndromes in Schizophrenia: A Cross-Cultural Study

**DOI:** 10.1155/2003/304095

**Published:** 2003-01-01

**Authors:** H. Ihara, G. E. Berrios, P. J. McKenna

**Affiliations:** ^1^Department of PsychiatryUniversity of CambridgeHills RoadCambridgeUK; ^2^Cambridge Psychiatric Rehabilitation ServiceFulbourn HospitalCambridgeUK

**Keywords:** cognitive dysfunction, dysexecutive syndrome, cross-cultural study, negative syndrome, schizophrenia

## Abstract

This paper examined the relationship between the ‘negative syndrome’ (NS) and the neuropsychological ‘dysexecutive syndrome’ (DES) in schizophrenia. The study also examined whether any relationship that exists between the NS and the DES holds equally for British and Japanese subjects. We compared 26 Japanese with 17 British schizophrenic patients, divided into ‘mild’ and ‘severe’ NS groups, on the basis of performance on neuropsychological tests, including the ‘Behavioural Assessment of Dysexecutive Syndrome’ (BADS). We found that patients with severe NS showed more everyday executive deficits than those with mild NS. The severity of NS was correlated with executive competence. The association between NS and the BADS performance was closer than that between NS and other conventional executive measures. These findings were not influenced by cultural differences between Japanese and British subjects, and, hence, suggested the existence of culture-neutral neurobehavioural processes.

## 1. Introduction

The underling mechanisms of pervasive deterioration in schizophrenia are important for a number of reasons. For example, the development of the deterioration may be seminal in distinguishing schizophrenia from other psychoses. On the one hand, a diagnosis of schizophrenia still depends, to some extent, on the cross-sectional analysis of conspicuous psychotic symptoms, such as delusions and hallucinations, collectively called 'positive symptoms' [9,19]. From a longitudinal viewpoint, on the other hand, such highly noticeable but commonly transient symptoms yield to less marked but persistent ones as follows: poor motivation, social withdrawal, blunted affect, slowness in thought process, and lack of fluency. These are called 'negative' symptoms. After exacerbation and remission of positive symptoms, a considerable proportion of patients fall into a state of severe deterioration with prominent negative symptoms. This state is called 'negative syndrome'.

Neurocognitive perspectives have attached greater importance to negative syndrome than positive one, due to the fact that schizophrenia was first viewed as a cognitive disorder. Kraepelin [21] named this psychosis 'dementia praecox', viewing cognitive deficits as irreversible and progressive. Since then, clinicians have been aware of a resemblance between cognitive deterioration in schizophrenia and the behavioural characteristics of frontal lobe disorder. For example, Frith [12] argues that a disorder of willed action may underlie behavioural characteristics common to both schizophrenic patients and frontal lobe patients, such as lack of volition, perseveration and behaviour elicited by

ISSN 0953-4180/03/$8.00 © 2003 – IOS Press. All rights reserved irrelevant external stimuli. This pattern of deficits can be understood as an impairment of attentional control at the level of Shallice's [34] 'supervisory system'. In Baddeley's model of working memory [4], this system is analogous to the 'central executive', the impairment of which causes 'dysexecutive syndrome' (DES). DES is a more functional characterisation of the so-called 'frontal lobe syndrome'. With the aim of predicting everyday problems arising from the DES, Wilson et al. [41] devised a neuropsychological test battery, the Behavioural Assessment of Dysexecutive Syndrome (BADS). Administering the BADS to schizophrenic patients, Evans et al. [11] found impairment on most of the dysexecutive tests. However, the relationship between negative syndrome and performances on the BADS in schizophrenia has not yet been examined.

The present study therefore attempts to fill the gap between 'negative syndrome' (NS) and BADS-defined DES in schizophrenia. The advantage of employing the BADS as a test of DES is its 'ecological validity' in terms of whether a test predicts a particular pattern of behaviour that is found in everyday situations. Conventional neuropsychological tests of executive functioning have so far measured objectively one's problem-solving abilities in impoverished experimental situations. However, it is questionable whether performance on these tests accurately predicts performance in daily living. These tests are not necessarily sufficient to contribute to the understanding of cognitive and behavioural deficits in 'everyday' situations [27, 36]. Building on criticism of traditional frontal tests, the BADS was designed to include the following characteristics: task initiation prompted by subjects themselves rather than by the examiner; tests requiring subjects to organise their behaviour for a relatively lengthy period of time; and tests requiring subjects to set priorities in the completion of two or more competing tasks. For example, the Modified Six Elements Test, one of the six sub-tests of the BADS, requires subjects to plan, organise and monitor their activities. They are asked to perform six tasks in ten minutes while obeying certain rules. The original version of this test was devised by Shallice and Burgess [35] to test the adequate functioning of a supervisory attentional system. This sort of task requirement is considered to be analogous to everyday executive abilities.

We also tested whether the association between NS and BADS would, if significantly close, survive correction for socio-cultural factors. This study was carried out as part of a cross-cultural research project investigating schizophrenia in terms of a number of neuropsychological and symptomatological variables. Its aim was to test whether schizophrenic patients with symptomatological NS from two distinctly different populations show similar neuropsychological DES. Indeed, international studies [42,43] have established groups of similar characteristics across a variety of Western and non-western cultures at the level of symptoms. In neuro-cognitive physiology, Allen et al. [3] demonstrated a similar pattern of anti-saccadic eye movements and attentional asymmetry in schizophrenia in three Pacific populations. Bobes et al. [5] also found comparable abnormalities of event-related potential N400 in Cuban and Chinese patients. We [18] have already reported that, in spite of major cultural differences, Japanese and British schizophrenic patients do not differ much in terms of their neuropsychological dysexecutive profiles. These studies, however, remain incomplete in that they can only demonstrate resemblance at a single level: symptomatic, neurophysiological or neuropsychological. We will take up the question as to whether the association between neuropsychological and symptomatic levels, i.e., BADS-defined DES and clinically-rated NS, is similar in schizophrenic patients from different cultures. If the association is similar, then the case for a common cognitive-symptomatic pathology in schizophrenia will be buttressed.

## 2. Methods and materials

### 2.1. Subjects

A total of 43 subjects participated in this study: 26 Japanese schizophrenic patients and 17 British schizophrenic patients.

The Japanese patients with schizophrenia who participated in this study were recruited from the following hospitals: Muroi Hospital, Oyama Fujimidai Hospital and Nasu Hillside Hospital, all of which lie in Tochigi Prefecture, approximately 100 km north of Tokyo, and from National South Hanamaki Hospital in Iwate Prefecture 600 km north of Tokyo. All the Japanese patients were being treated with typical neuroleptics at the time of neuropsychological assessment.

The British patients with schizophrenia who took part in the study were recruited from a psychiatric rehabilitation service in Cambridge. They were all Caucasians and native English speakers. The National Adult Reading Test (NART) [28] was administered and provided a measure of premorbid intellectual functioning in British patients suspected of suffering from intellectual deterioration. Their scores on the NART and the duration and type of their education indicated that they were considered to be of average premorbid intelligence. 12 patients were taking clozapine and 5 typical neuroleptics.

All patients underwent testing while clinically stable. Semi-structured diagnostic interviews revealed that all Japanese and British patients met the criteria for schizophrenia according to the Diagnostic and Statistical Manual of Mental Disorders: Fourth Edition (DSM-IV) [1] and the International Classification of Diseases (ICD-10) [44]. For British patients, research diagnosis was reinforced by a consultant (PJM) and ward colleagues following a review of medical records, a formal diagnostic conference and an extended period of clinical observation.

Patients with co-morbidity of other mental illnesses or a history of electroconvulsive therapy within the previous two years were excluded. All subjects were screened for a history of head injury followed by loss of consciousness, substance abuse, general somatic conditions that might affect neuropsychological performance, and learning disorders. In cases of those taking anticholinergic agents, doses were below the level affecting memory test performance in schizophrenia, i.e., the range 5–15 mg procyclidine daily [37]. We obtained a written form of consent from all of the participants after the procedure had been fully explained. We also obtained ethical approval from both the Cambridge Local Research Ethics Committee and the Clinical Management Committee of each Japanese hospital.

Between the two groups with schizophrenia, we found no significant statistical differences in age ($t = 1.445, p = 0.156$), duration of education ($t = -0.203$, $p = 0.840$), duration of illness ($t = 0.303, p = 0.763$), age of onset ($t = 1.457$, $p = 0.153$) and neuroleptic doses of chlorpromazine equivalents ($t = -0.722$, $p = 0.474$) [10,20].

### 2.2. Clinical symptom rating

Symptomatological evaluations for the schizophrenic groups were made according to semi-structured clinical interviews and standardised rating scales for the Positive and Negative Syndrome Scale (PANSS) [19] by an English-speaking Japanese psychiatrist (H.I.) and using the patients' case records. We found no significant difference in positive syndrome ($t = -1.881$, $p = 0.067$) and negative syndrome ($t = -1.520$, $p = 0.140$).

The patients were divided into 'mild' negative syndrome (mild NS) and 'severe' negative syndrome (severe NS) groups by segregating in terms of a cut-off score of 19/20 of the NS score in the PANSS. Table 1 provides demographic and baseline variables for the Japanese patients with severe NS and with mild NS, and the British patients with severe NS and with mild NS. Two-way ANOVA revealed that NS severity, nationality and the interaction of NS severity and nationality did not prove significant in terms of age, education, duration of illness, age of onset and neuroleptic dose of chlorpromazine equivalents. Patients with severe NS showed a significantly more severe positive syndrome than those with mild NS ($df = 1, 42$; $F = 10.057$; $p = 0.003$). But neither nationality, nor the interaction between NS severity and nationality were significant in relation to positive syndrome.

### 2.3. Neuropsychological assessment

The extended battery of neuropsychological tests of executive functioning applied to all patients, including: the Raven Advanced Progressive Matrices [30]; the Verbal Fluency Test (VFT) [25]; the Trail Making Test (TMT) [31]; the Stroop Colour Word Test (SCW) [38]; and the Behavioural Assessment of Dysexecutive Syndrome (BADS) [41]. We have given a brief account of the application of these tests to Japanese subjects [18]. A full explanation is unnecessary due to their clinical popularity. More detailed descriptions can be obtained from the literature shown above.

#### 2.3.1. *Raven advanced progressive matrices* (*RAPM*)

This 'culture-free' test of general fluid intelligence consists of 12 visually-based pattern-reasoning tests arranged in order of complexity. The RAPM seems less susceptible to cultural factors than other tests such as the Wechsler Adult Intelligent Scale [39] and NART.

#### 2.3.2. *Verbal fluency test* (*VFT*)

This test asks subjects to produce orally as many words as possible beginning with a particular letter in sixty seconds. In this project, the initial letters 'F', 'A' and 'S' are used for British subjects, whereas letters 'H', 'A' and 'S' are applied to Japanese individuals. The rationale for comparison between English VFT and its Japanese equivalent is provided by the following: the total number of words starting with the letter 'F', 'A' or 'S' comprise 23.1% of the entire English vocabulary (F: 4.9%; A: 5.3%; S: 12.9%)(Oxford English Dictionary), whereas the total number of words begin-

Table 1
Demographic and baseline variables of the Japanese schizophrenic patients with severe negative syndrome (NS) and with mild NS, and the British schizophrenic patients with severe NS and with mild NS

| | Japanese Schizophrenic Patients with severe NS ($n = 13$) | Japanese Schizophrenic Patients with mild NS ($n = 13$) | British Schizophrenic Patients with severe NS ($n = 9$) | British Schizophrenic Patients with mild NS ($n = 8$) |
|---|---|---|---|---|
| Age (yr) 44.2 (9.7) | 42.2 (11.9) | 36.9 (8.1) | 40.9 (8.2) | |
| Sex (F:M) | 8: 5 | 10: 3 | 2: 7 | 1: 7 |
| Duration of illness (yr) | 17.6 (8.9) | 13.6 (11.6) | 14.2 (9.3) | 15.3 (6.8) |
| Age of onset | 26.8 (10.7) | 28.5 (8.9) | 22.7 (5.7) | 25.6 (7.5) |
| Medication in CPZ equivalents | 638.5 (331.1) | 424.6 (588.8) | 521.7 (416.6) | 768.8 (497.8) |
| Anticholinergics | 13: 0 | 11: 2 | 6: 3 | 4: 4 |
| Duration of education (yr) | 12.2 (2.5) | 12.6 (2.2) | 11.8 (1.6) | 13.4 (2.6) |
| NART | | 105.7 (15.2) | 107.6 (12.3) | |
| Positive syndrome | 16.2 (5.1) | 11.5 (5.1) | 19.4 (3.9) | 114.4 (5.4) |
| Negative syndrome | 23.1 (3.1) | 14.9 (2.6) | 27.1 (3.4) | 15.9 (2.5) |

Note. NART = National Adult Reading Test [28].

ning with the letter 'H', 'A' or 'S' account for 26.3% of the whole Japanese vocabulary (H: 9.8%; A: 3.2%; S: 13.3%) (Shinchosha Modern Japanese Dictionary). We used the total number of all correct words, produced by subjects, as their score.

*2.3.3. Trail making test* (*TMT*)

All subjects performed both Parts A and B of the TMT. For Japanese subjects, Hiragana characters are used in Part B, according to the dictionary arrangement of the Japanese syllabary. Each Japanese character is placed on the corresponding letter from the Roman alphabet in the correct order. For example, the first *Hiragana* character あ, (pronunciation: short 'a') is placed exactly in the same place as 'A', and the second letter い (pronunciation: short 'i') in the same place as 'B'.

*2.3.4. The stroop colour word test* (*SCW*)

This consists of two tasks, in both of which subjects are given a page with a series of 112 colour words printed in different colours of ink. The number of correct responses in the second task (Colour Word Task) is scored. A Japanese version with the same number of colour words printed in different colours was administered to Japanese subjects. The colour words in the Japanese versions were similar in number of syllables to their English counterparts.

*2.3.5. Behavioural assessment of dysexecutive syndrome* (*BADS*)

This consists of six sub-tests. From each a 'summary profile score' is obtained (with a maximum of 4 and minimum of 0) and these are added up to produce an overall score (out of 24). We applied the same procedure to both Japanese and British participants, although instruction to Japanese subjects was spoken in Japanese. In the *Zoo Map*, Japanese subjects were given Japanese maps in which all places and relevant instructions were written in Japanese. They were also given a Japanese instruction sheet in which all instructions were written in Japanese in the *Modified Six Elements*.

*2.4. Analyses*

We used the SPSS V8 Package for descriptive and inferential statistical analyses. Cases with non-missing data were used, on the assumption that data were missing at random.

The statistical analyses addressed the following basic predictions: (1) patients with severe NS would perform more poorly on neuropsychological tests in general and BADS in particular than those with mild NS; (2) neither nationality nor the interaction between nationality and severity would have a significant impact on neurobehavioural measures; (3) the severity of NS would be correlated with everyday executive measures even after removing the effects of age, education, duration of illness and nationality; (4) the severity of everyday executive impairment would independently contribute to the severity of NS, significantly enough to survive correction for other aspects of executive dysfunction.

To assess the first and second predictions, neuropsychological measures were submitted to an analysis of variance (ANOVA) using two between-subject factors: severity of NS (severe NS versus mild NS) and nationality (Japanese versus British). To assess the third prediction, we obtained partial correlations, for both Japanese and British schizophrenic patients. These cor-

Table 2
Average scores (and standard deviations) for the Japanese schizophrenic patients with severe negative syndrome (NS) and with mild NS, and the British schizophrenic patients with severe NS and with mild NS on the RAPM, the VFT, the TMT Part A and B, the SCW, and the BADS sub-tests and BADS Profile Score

| | Japanese Schizophrenic Patients with severe NS ($n = 13$) | Japanese Schizophrenic Patients with mild NS ($n = 13$) | British Schizophrenic Patients with severe NS ($n = 9$) | British Schizophrenic Patients with mild NS ($n = 8$) |
|---|---|---|---|---|
| RAPM | 4.62 (1.66) | 7.62 (2.72) | 4.67 (1.41) | 7.75 (1.98) |
| VFT | 29.0 (10.3) | 35.8 (17.2) | 30.7 (8.0) | 45.8 (14.0) |
| TMT A | 44.6 (28.7) | 38.6 (10.1) | 52.1 (14.2) | 47.4 (17.7) |
| TMT B | 172.5 (120.7) | 115.9 (47.7) | 192.9 (82.4) | 116.3 (53.3) |
| SCW | 73.8 (21.3) | 89.4 (22.4) | 74.0 (21.9) | 85.0 (20.6) |
| BAS | | | | |
| Rule shift cards | 1.92 (1.38) | 2.92 (1.55) | 2.22 (1.20) | 3.71 (0.76) |
| Action programme | 2.62 (1.45) | 3.23 (1.54) | 2.56 (1.67) | 3.57 (0.53) |
| Key search | 1.15 (1.21) | 2.54 (1.20) | 1.78 (1.20) | 3.00 (1.31) |
| Temporal judgement | 1.08 (1.04) | 1.77 (0.73) | 1.22 (1.09) | 1.86 (1.21) |
| Zoo map | 2.54 (1.05) | 3.00 (0.82) | 1.78 (1.09) | 2.38 (1.19) |
| Modified six elements | 1.61 (1.45) | 3.23 (1.09) | 2.00 (1.22) | 3.43 (1.13) |
| Profile score | 10.92 (4.05) | 16.69 (4.53) | 11.56 (4.45) | 18.14 (2.19) |

Note. RAPM = Raven Advanced Progressive Matrices [30]; VFT = Verbal Fluency Test [25]; TMT = Trail Making Test [31]; SCW = Stroop Colour Word Test [38]; BADS = Behavioural Assessment of Dysexecutive Syndrome [41].

relations were between neuropsychological measures, on the one hand, and psychopathological variables, on the other, after the effects of age, education, duration of illness and nationality were discounted. To assess the fourth prediction, we performed a series of multiple linear regression analyses. These analyses were concerning the association between the BADS profile score as an independent variable and NS as a dependent variable. They were for the purpose of obtaining information unavailable from partial correlations, i.e. the independent contribution of everyday problem-solving competence and other cognitive variables to the severity of NS. As the psychometric testing of frontal executive functions are known to be effected by baseline intellectual variables, age and education were entered first into the regression model. The duration of illness and nationality were also entered to minimise the effect of factors irrelevant to the prediction. The scores of the RAPM [30], the VFT, the TMT Part B and the SCW were then added in turn to ascertain whether the association between the BADS profile score and NS survived correction for these. The purpose of such forced entry of traditional measures of fluid intelligence and frontal executive functions was to define the degree to which covariance with cognitive domains other than everyday executive competence might underlie observed relationships. We thus assessed the possibility that BADS profile scores include an 'ecological' domain that is not completely explained by conventional 'non-ecological' tests but that is nevertheless important in understanding NS. We finally performed the same series of multiple linear regression analyses, using the score on the Modified Six Elements, instead of the BADS profile score, as an independent variable. This is due to the aforementioned importance of this BADS sub-test to the assessment of executive deficits in a way that reflects the difficulties patients have in daily life.

## 3. Results

Table 2 shows the results of the neuropsychological tests for the four groups and Table 3 those of an ANOVA. It was confirmed that patients with severe NS performed significantly worse than those with mild NS on a test of fluid intelligence (RAPM) and conventional executive tests (VFT, TMT B). The inter-group difference on the TMT A and SCW did not reach the level of statistical significance. The ANOVA of the BADS profile score revealed highly significant differences between severe and mild NS groups. The ANOVA of subtests in the BADS battery showed that patients with severe NS performed significantly worse than those with mild NS on five out of six sub-tests. In contrast to the severity of NS, nationality did not have a significant impact on the performance of neuropsychological tests, irrespective of which cognitive domains were assessed by different tests. Furthermore, the interaction between NS severity and nationality was not significant in any test.

Table 4 shows that the RAPM as a test of fluid intelligence was correlated significantly with a wide spectrum of negative syndrome as well as with the overall NS score, but not with the positive symptom score. As

Table 3
ANOVA results upon neuropsychological results, using two between-subject factors: nationality (Japanese versus British) and negative syndrome condition (high negative versus low negative)

| | Severity of NS | | Nationality | | Severity of NS | ×Nationality |
|---|---|---|---|---|---|---|
| | F (1, 38) | P | F (1, 38) | P | F (1, 38) | P |
| RAPM | 23.05 | <0.001** | 0.11 | 0.743 | 0.06 | 0.802 |
| VFT | 8.51 | 0.006** | 2.78 | 0.104 | 1.60 | 0.214 |
| TMT A | 0.41 | 0.496 | 2.18 | 0.148 | 0.08 | 0.780 |
| TMT B | 8.08 | 0.007** | 0.005 | 0.944 | 0.49 | 0.488 |
| SCW | 3.83 | 0.058 | 0.06 | 0.811 | 0.07 | 0.794 |
| BADS | | | | | | |
| Rule shift cards | 8.66 | 0.006** | 1.66 | 0.206 | 0.34 | 0.565 |
| Action programme | 3.21 | 0.081* | 0.10 | 0.760 | 0.19 | 0.663 |
| Key search | 10.83 | 0.002** | 1.88 | 0.179 | 0.04 | 0.839 |
| Temporal judgement | 4.36 | 0.044* | 0.14 | 0.716 | 0.008 | 0.929 |
| Zoo map | 3.81 | 0.058 | 3.42 | 0.072 | 0.267 | 0.608 |
| Modified six elements | 14.61 | <0.001** | 0.535 | 0.469 | 0.055 | 0.816 |
| Profile score | 22.56 | <0.001** | 0.641 | 0.428 | 0.099 | 0.755 |

$^{*}p < 0.05$.

$^{**}p < 0.01$.

Note. RAPM = Raven Advanced Progressive Matrices [30]; VFT = Verbal Fluency Test [25]; TMT = Trail Making Test [31]; SCW = Stroop Colour Word Test cite38; BADS = Behavioural Assessment of Dysexecutive Syndrome [41].

far as traditional frontal tests were concerned, significant correlations were observed between the overall NS score and two psychometric indices, i.e. the VFT and the TMT B. These tests were also correlated with a few individual negative symptoms, such as poor rapport and difficulty in abstract thinking. By contrast, the SCW, although considered a test of frontal executive functioning, was not correlated with any symptomatic indices. As for the BADS, the profile score and scores on two sub-tests, i.e. the Zoo Map and the Modified Six Elements, were correlated significantly with the overall NS score. With regard to negative syndrome, poor rapport, abstract thinking difficulty, lack both of spontaneity and of conversational flow, and stereotyped thinking were closely associated with major neuropsychological variables. However, other symptoms, such as blunted affect, emotional withdrawal and passive/apathetic social withdrawal, were not always correlated with scores on executive tests.

The stepwise regression of the NS overall score, using the BADS profile score and additional variables, yielded only two significant predictor variables: the BADS profile score, and nationality (Table 5). The standardised partial regression coefficient for the BADS was $-0.486$. When the RAPM as a test of fluid intelligence was forced to enter the regression, this was revealed to be a significant predictor (standardised partial regression coefficient $-0.455$) of the severity of NS, with the BADS losing statistical significance. Thus the relationship between the BADS and NS did not survive correction for the RAPM. However, the forced entry of three traditional measures of frontal executive functioning did not have much influence on the relationship. Even after the VFT, the TMT B and the SCW entered the regression, the BADS remained a significant predictor. Moreover, the independent contribution of the VFT and the SCW to the prediction of NS severity did not reach the level of $p < 0.05$.

We finally examined the effect of the Modified Six Elements, one of the BADS' six sub-tests, on the severity of NS (Table 6). The same series of multiple linear regression analyses was performed. That was the stepwise regression of the NS overall score, using the Modified Six Elements score and additional variables as independent variables, followed by the forced entry of the four traditional tests. The Modified Six Elements score was revealed to be a significant predictor variable, with a standardised partial regression coefficient of $-0.452$. The relationship between the Modified Six Elements and NS severity survived correction for the RAPM, the VFT, the TMT and the SCW. Thus, difficulties in carrying out multiple tasks in a limited time period, which is the cognitive function assessed by the Modified Six Elements, had a significantly close relationship with NS. Besides, this relationship survived correction for cognitive domains assessed by other cognitive tests.

## 4. Discussion

These data lead to four major findings: (1) schizophrenic patients with severe NS demonstrate

Table 4
Partial correlations between negative syndrome and cognitive variables, removing effects of age, education, the duration of illness and nationality

| | N1 | N2 | N3 | N4 | N5 | N6 | N7 | NS | PS |
|---|---|---|---|---|---|---|---|---|---|
| RAPM | −0.512** | −0.450** | −0.470** | −0.453** | −0.577** | −0.612** | −0.417** | −0.615** | −0.261 |
| VFT | −0.391 | −0.256 | −0.382* | −0.083 | −0.544** | −0.270 | −0.328* | −0.396* | −0.391* |
| TMT A | 0.303 | 0.240 | 0.312 | 0.156 | 0.343* | 0.198 | 0.050 | 0.297 | 0.143 |
| TMT B | 0.526** | 0.433** | 0.520** | 0.447 | 0.506** | 0.502** | 0.254 | 0.553** | 0.020 |
| SCW | −0.187 | −0.076 | −0.282 | −0.205 | −0.287 | −0.257 | −0.206 | −0.263 | −0.102 |
| BADS | | | | | | | | | |
| Rule shift cards | −0.266 | −0.060 | −0.294 | −0.088 | −0.437** | −0.212 | −0.236 | −0.267 | −0.143 |
| Action programme | −0.133 | −0.256 | −0.210 | −0.237 | −0.415** | −0.134 | −0.075 | −0.226 | −0.141 |
| Key search | −0.091 | −0.029 | −0.260 | −0.094 | −0.408* | −0.270 | −0.242 | −0.279 | −0.320 |
| Tempo-ral judge-ment | −0.226 | −0.150 | −0.242 | −0.312 | −0.250 | −0.123 | −0.077 | −0.315 | −0.178 |
| Zoo map | −0.195 | −0.211 | −0.342* | −0.266 | −0.413* | −0.322* | −0.316* | −0.357* | −0.111 |
| Modified six elements | −0.235 | −0.145 | −0.467** | −0.255 | −0.450** | −0.263 | −0.464** | −0.445** | −0.437** |
| Profile score | −0.294 | −0.200 | −0.472** | −0.315 | −0.623** | −0.340* | −0.371* | −0.486** | −0.358** |

*$p < 0.05$.

**$p < 0.01$.

Note. N1 = Blunted affect; N2 = Emotional withdrawal; N3 = Poor rapport; N4 = Passive/apathetic social withdrawal; N5 = Difficulty in abstract thinking; N6 = Lack of spontaneity and flow of conversation; N7 = Stereotyped thinking; NS = Negative syndrome; PS = Positive syndrome; RAPM = Raven Advanced Progressive Matrices [30]; VFT = Verbal Fluency Test [25]; TMT = Trail Making Test [31]; SCW = Stroop Colour Word Test [38]; BADS = Behavioural Assessment of Dysexecutive Syndrome [41].

more severe deficits in terms of everyday executive problems as assessed by the BADS, as well as in other cognitive domains as assessed by the RAPM, the VFT and the TMT Part B, than those with mild NS; (2) neither cultural differences between Japanese and British nor the interaction between the differences and NS severity influenced performances on neuropsychological tests in general, and on the BADS in particular; (3) the severity of NS was correlated with everyday executive competence and, in particular, with the subjects' abilities to organise and monitor their own behaviour, as assessed by the Zoo Map and the Modified Six Elements; (4) the association between everyday executive functioning and NS was close, and included that part of the cognitive domain that was not fully registered by conventional executive tests.

These findings indicate that impairment of everyday executive competence is closely associated with NS. Although some prior studies [6,7,17,24,40] showed that NS is associated with frontal executive deficits, these studies administered conventional executive tests only. For example, even the Wisconsin Card Sorting Test (WCST), although considered the *sine qua non* of frontal lobe tests [13,23], is a highly multidimensional task. This fact casts serious doubt upon the recent application of conventional executive tests in relation to the neuropsychology of DES [14,22]. In consequence, despite observation of phenomena resembling NS and DES, empirical findings in this area are not always consistent. In fact, Morrison-Stewart et al. [26] did not find evidence of a relationship between negative syndrome and executive functions as assessed by the WCST and word and design fluency tests. This might, however, be due to the limitations of currently available psychometric techniques in specifying such functions. By contrast, this study, by administering the BADS, illuminates the existence of specific executive domains that are not fully assessed by traditional frontal tests but that are nevertheless relevant to understanding NS.

A potential criticism of the above argument is that degrees of tardive dyskinesia, akathisia and drug-induced Parkinsonism were not controlled properly. This leads to the possibility that the differences between patients with severe NS and those with mild NS simply reflected differing degrees of neurological motor disorders rather than of cognitive competence. This potential criticism is, however, unlikely to be valid in that everyday executive tasks on the BADS do not have a significant speed component compared with conventional executive tests. On the other hand, the TMT A and the SCW, although they have a much greater speed component than sub-tests of the BADS, revealed few inter-group differences. Furthermore, even after excluding the effect of executive tests with a substantial speed component, such as the VFT, the TMT B and the SCW, the relationship between everyday DES and NS remained significant.

Unfortunately we cannot completely exclude the possibility that the relationship between everyday DES and NS could be interpreted in terms of a general level of cognitive impairment rather than specific executive deficits. Indeed, the relationship between the BADS

Table 5
Stepwise regression analysis of the negative syndrome using BADS, age, education, the duration of illness and nationality, followed by the forced entry of the RAPM, the VFT, the TMT B and the SCW

| Regression Model | R, F and Beta | P |
|---|---|---|
| Stepwise Regression Model of the NS | R=0.550, F=8.462 | 0.001** |
| Variables in the Equation | | |
| BADS | Beta= $-0.486$ | 0.001** |
| Nationality | Beta=0.289 | 0.037* |
| Variables excluded from the Equation | | |
| Age | – | n.s. |
| Education | – | n.s. |
| Duration of illness | – | n.s. |
| Forced Entry of the RAPM | R=0.674, F=10.518 | <0.001** |
| Variables in the Equation | | |
| BADS | Beta= $-0.249$ | 0.084 n.s. |
| Nationality | Beta= 0.276 | 0.028* |
| RAPM | Beta= $-0.455$ | 0.002** |
| Forced Entry of the VFT | R=0.598, F=7.061 | 0.001** |
| Variables in the Equation | | |
| BADS | Beta= $-0.394$ | 0.008* |
| Nationality | Beta=0.336 | 0.016* |
| VFT | Beta= $-0.258$ | 0.078 n.s. |
| Forced Entry of the TMT B | R=0.630, F=8.330 | <0.001** |
| Variables in the Equation | | |
| BADS | Beta= $-0.324$ | 0.029* |
| Nationality | Beta=0.264 | 0.044* |
| TMT B | Beta=0.347 | 0.020* |
| Forced Entry of the SCW | R=0.550, F=5.458 | 0.003** |
| Variables in the Equation BADS | Beta= $-0.483$ | 0.003** |
| Nationality | Beta=0.289 | 0.041* |
| SCW | Beta= $-0.007$ | 0.960 n.s. |

$^{*}p < 0.05$.

$^{**}p < 0.01$.

Note. RAPM = Raven Advanced Progressive Matrices [30]; VFT = Verbal Fluency Test [25]; TMT = Trail Making Test [31]; SCW = Stroop Colour Word Test [38]; BADS = Behavioural Assessment of Dysexecutive Syndrome [41].

and NS did not survive correction for the score of a test of general intelligence (RAPM). However, the Modified Six Elements remained a significant predictor of the NS overall score, even after the RAPM entered the regression. Therefore, it seems likely that some specific aspects of executive deficits (e.g. difficulties in carrying out multiple tasks) rather than general intellectual impairment could be associated with the level of NS. But, this issue should be tested on the basis of a much larger sample of schizophrenic patients with minimal intellectual decline.

We should clarify the issue of the difference in the two nationalities in terms of level of NS. In fact, nationality was a significant predictor of the NS overall score in the regression analyses. The NS level was not completely controlled between the Japanese patients and the British patients, although the difference did not reach the level of statistical significance. In our opinion, this finding arose merely from a sampling bias in terms of the milder level of NS in the Japanese group.

This study has further limitations. All patients were receiving medication when they underwent neuropsychological and symptomatic assessment. Furthermore, antipsychotic and anticholinergic medication for each patient was different in terms of type and dose. To eliminate such differences would have been impracticable by virtue of cross-cultural differences in pharmacotherapy. For example, clozapine has not yet been authorised in Japan, whereas 12 British patients were taking this atypical antipsychotic drug. For generalisation from the present results, a more rigorous research design is required in order to control the effects of medication on clinical features and neuropsychological functions.

The close association between BADS-based executive impairment and the severity of NS suggests that BADS scores are representative of daily problems experienced by schizophrenic patients and, potentially, provide useful information in understanding NS. For example, among seven individual symptoms of PANSS-rated NS, N3 (poor rapport), N5 (abstract thinking dif-

Table 6
Stepwise regression analysis of the negative syndrome using the Modified Six Elements, age, education, the duration of illness and nationality, followed by the forced entry of the RAPM, the VFT, the TMT B and the SCW

| Regression Model | R, F and Beta | P |
|---|---|---|
| Stepwise Regression Model of the NS | R=0.520, F=7.224 | 0.002** |
| Variables in the Equation | | |
| Modified Six Elements | Beta= $-0.452$ | 0.002** |
| Nationality | Beta=0.291 | 0.041* |
| Variables excluded from the Equation | | |
| Age | – | n.s. |
| Education | – | n.s. |
| Duration of illness | – | n.s. |
| Forced Entry of the RAPM | R=0.684, F=11.153 | <0.001** |
| Variables in the Equation | | |
| Modified Six Elements | Beta= $-0.265$ | 0.046*. |
| Nationality | Beta=0.279 | 0.024* |
| RAPM | Beta= $-0.482$ | 0.001** |
| Forced Entry of the VFT | R=0.585, F=6.598 | 0.001** |
| Variables in the Equation | | |
| Modified Six Elements | Beta= $-0.363$ | 0.013* |
| Nationality | Beta=0.343 | 0.015* |
| VFT | Beta= $-0.289$ | 0.048* |
| Forced Entry of the TMT B | R=0.639, F=8.718 | <0.001** |
| Variables in the Equation | | |
| Modified Six Elements | Beta= $-0.323$ | 0.019* |
| Nationality | Beta=0.265 | 0.041* |
| TMT B | Beta=0.392 | 0.005** |
| Forced Entry of the SCW | R=0.530, F=4.948 | 0.005** |
| Variables in the Equation | | |
| Modified Six Elements | Beta= $-0.425$ | 0.005** |
| Nationality | Beta=0.283 | 0.048* |
| SCW | Beta= $-0.106$ | 0.459 n.s. |

$^{*}p < 0.05$.

$^{**}p < 0.01$.

Note.RAPM = Raven Advanced Progressive Matrices [30]; VFT = Verbal Fluency Test [25]; TMT = Trail Making Test [31]; SCW = Stroop Colour Word Test [38].

ficulty), N6 (lack of spontaneity and flow of conversation), and N7 (stereotyped thinking) were closely correlated with scores of a number of BADS sub-tests.

N6 is characterised by diminished fluidity and productivity in the verbal-interactional process. Patients with N7 have difficulty in shifting from one idea to another and conversation is dominated by the constant repetition of fixed ideas or limited phrases. Decreased fluidity, as well as spontaneity and flexibility, as rated in conversation (N6) and in thinking (N7), are replicated in patients' performances on the BADS. For example, in the Modified Six Elements, patients with severe NS tended to spend too much time on particular sub-tasks, despite the rule stating that they attempt at least something on each of the six sub-tasks. Once they became engrossed in one sub-task, they failed to switch to another. On the Zoo Map test, patients' decreased flexibility in thinking manifested itself in an inability to inhibit a response pattern. For example, even after finding that the route they had taken was wrong, some patients with severe NS tried to take the same route again, instead of trying others. Once they formed one stereotyped route pattern, they tended to repeat the same error. Although they recognised the strategy they were taking was wrong, it seemed they did not reflect upon their actions.

Such lack of fluidity and productivity in thinking and conversation may be due in part to patients' difficulty in abstract thinking as rated in terms of N5. On the PANSS, N5 is rated based on responses to questions on perceived similarities between objects and proverb interpretation. N5 may be related to difficulties in proceeding beyond concrete thinking in a problem-solving task. For example, in the Action Programme, patients with severe NS were unable to take on board the fact that objects without apparent connection were being presented in the situation as clues. They tried to draw out the cork with the metal rod and found it too short.

Nevertheless, they did not place these objects inside a conceptual frame of reference and, in consequence, failed to think of an alternative use for the rod. Bound to the immediate experience of given environmental information, they were unable to shift voluntarily from one aspect of a situation to another.

These findings are consistent with the cognitive-anatomic hypothesis that the simultaneous occurrence of NS and DES in schizophrenia is related to dysfunction in the frontal cortex and its allied structures. Due however to the lack of direct data from neuroimaging, our functional-anatomic discussion is confined to mere speculation. Even so, other localising hypotheses have less empirical support. A considerable body of evidence has accumulated in favour of a close association between frontal lobe damage and impaired performance on executive tests [8,29,32,35]. As for the BADS, there are few empirical data in terms of functional anatomy of performance on each of the six sub-tests. Administering the BADS, Evans et al. [11] found that the performance pattern of schizophrenic patients is similar to that of patients with brain injury. In their study, the majority of brain-injured patients had anterior or frontal/temporal damage, although they were heterogeneous in terms of aetiology and severity of injury. Further research is necessary to specify the functional anatomy of the association between NS and everyday executive impairment in schizophrenia.

Both the first and second stepwise regression analyses of the NS severity showed patients' nationality as a significant predictor. The first model accounted for 30.3% of the variance in NS with the BADS and nationality as significant contributors, whereas the second model accounted for 27.0% of the variance in NS with the Modified Six Elements and nationality as significant contributors. This may be consequent upon degrees of difference of NS between Japanese and British schizophrenic groups ($t = -1.520$, $p = 0.140$), although not statistically significant. In addition to sample selection bias, cultural variation in the severity of NS is not entirely surprising. Granted such a cross-cultural symptomatic difference is valid, everyday executive impairment can be said to predict independently the severity of symptomatic NS.

We [18] have already reported that both Japanese and British groups of schizophrenic patients showed equally severe impairment on most executive tests including the BADS. In that study 18 Japanese and 22 British patients and 14 Japanese and 19 British control subjects were compared on the BADS and other measures of frontal executive functioning. Irrespective of cultural origin, similar neuropsychological dysexecutive profiles were found in both groups of schizophrenic patients.

Further, in this study, we have shown that a similar association between two levels of analysis, i.e. neuropsychological DES and symptomatic NS, is found in schizophrenic patients from two different cultures. This suggests that cognitive-symptomatic associations are relevant to understanding schizophrenia and may provide evidence of underlying culture-neutral neurobehavioural processes.

It is, however, true that the biological base of schizophrenia is said to be influenced by socio-cultural factors which can diversify the course of the disorder. This has been discussed by a number of authors. For example, Schooler and Caudill [33], who contrasted the symptom patterns of schizophrenic patients admitted to two public hospitals, one in Tokyo, Japan and the other in Maryland, USA. They found that the Japanese patients showed significantly more aggression, social withdrawal, apathy, and sleep disturbances than the American patients and that the American patients experienced significantly more hallucinations and bizarre ideas. Even so, this type of difference pertains to situations where socio-cultural factors are of great importance to the demarcation of normality and abnormality. Cross-cultural studies have so far largely explored the behavioural and symptomatic characteristics, rather than neurobiological bases, of mental illness. In consequence, maladaptive behaviour particular to the social environment has been by and large documented as if it were inherent to the very core of the disorder.

In order to achieve a balance between culture-related deviations and universally accepted abnormality, extending experimental psycho-physiological research on schizophrenia across cultures needs to be carried out. In Al-Issa's [2] opinion, such studies should be minimally influenced by culture and might be used as a baseline in comparing normal and schizophrenic subjects within, as well as across, cultures. But his proposals have rarely been implemented, due to the absence of a reliable physiological marker for schizophrenia and the difficulty of applying a complex experimental apparatus to cross-cultural studies.

Neuropsychological studies might allow for a possible modification of Al-Issa's proposed physiological experiment. Due to the relative portability of equipment for assessment, neuropsychological tests can now be applied in cross-cultural comparisons. These comparisons are important not only for providing psychometric information about mental illness, but also in reinforcing the analysis of cross-cultural diversity by calibrating such diversity from neutral perspectives.

## Acknowledgements

We thank all staff members of the Cambridge Psychiatric Rehabilitation Service, Muroi Hospital, Oyama Fujimidai Hospital, and Nasu Hillside Hospital who allowed us to examine patients under their care. We are also grateful to Professors Eric Chen and Seiji Yasumura for comments upon earlier drafts of this paper. We have received no external funding for this research.
